# COGNITIVE IMPAIRMENT AND SLEEP DIFFICULTIES OVER 10 YEARS IN A NATIONAL SAMPLE OF OLDER ADULTS

**DOI:** 10.1093/geroni/igz038.1696

**Published:** 2019-11-08

**Authors:** Rebecca Robbins, Amanda Sonnega, Robert W Turner II, Girardin Jean-Louis, Kenneth Langa

**Affiliations:** 1 NYU School of Medicine, New York, United States; 2 University of Michigan, Michigan, United States; 3 The George Washington University, Washington, District of Columbia, United States; 4 University of Michigan, Ann Arbor, Michigan, United States

## Abstract

Prior studies suggest that sleep difficulties (e.g., trouble falling asleep) may be associated with cognitive impairment. We used a large, nationally representative longitudinal survey of adults over the age of 50 in the US to examine the relationship between sleep difficulties and cognitive functioning. Generalized estimation equation (GEE) linear regression models were used to analyze data from the 2004-2014 waves of the Health and Retirement Study. We examined sleep difficulties and cognitive functioning within participants and across time (n=17,642). Sleep difficulty was measured as trouble falling asleep, nocturnal awakenings, and waking too early scored as 1= rarely/never, 2=sometimes, and 3=most of the time. A summary score indicated cognitive functioning (range 0-27). Models controlled for age, gender, race/ethnicity, marital status, education, chronic medical conditions, depressive symptoms, and body mass index (BMI). Compared to those with no sleep difficulties, those who reported difficulty falling asleep [“sometimes” OR=0.83,95%CI:0.71-0.96 and “most of the time” OR=0.79,95%CI: 0.64-0.98] and waking too early [“most of the time” OR=0.79,95%CI: 0.63-0.98] had worse cognitive functioning. Compared to those with no sleep difficulties, those who reported nocturnal awakenings [“most of the time” OR=1.29,95%CI:1.08-1.54] had higher cognitive functioning. Over time, lower cognitive function was more likely among those reporting difficulty falling asleep (OR=0.73,95%CI:0.54-0.97), nocturnal awakenings (OR=0.77,95%CI:0.61-0.97) and waking too early (OR=0.65,95%CI: 0.47-0.88). In this nationally representative, longitudinal sample of older US adults, we found that over time lower cognitive function was more likely among those who reported difficulty falling asleep, nocturnal awakenings, and waking too early.

